# Hesperidin Prevents Retinal and Plasma Abnormalities in Streptozotocin-Induced Diabetic Rats

**DOI:** 10.3390/molecules171112868

**Published:** 2012-11-01

**Authors:** Xiupu Shi, Sha Liao, Huijuan Mi, Changrun Guo, Dongli Qi, Fei Li, Chunfeng Zhang, Zhonglin Yang

**Affiliations:** 1 State Key Laboratory of Natural Products and Functions, China Pharmaceutical University, No. 24 Tongjiaxiang, Nanjing 210009, China; Email: shixiupu@yahoo.com.cn (X.S.); mihuijuan@yahoo.cn (H.M.); guochangrun@126.com (C.G.); dongliqi1983@gmail.com (D.Q.); lifeicpu@163.com (F.L.); 2 College of Life Sciences, Northwest University, Taibai Road 229, Xi’an 710069, China; Email: hisara126@126.com

**Keywords:** hesperidin, diabetic retinopathy, anti-angiogenic effect, aldose reductase activity, AGEs accumulation

## Abstract

Diabetic retinopathy is a complex disease that potentially involves increased production of advanced glycosylation end products (AGEs) and elevated aldose reductase (AR) activity, which are related with oxidative stress and inflammation. The aim of this study was to investigate the effects of hesperidin on retinal and plasma abnormalities in streptozotocin-induced diabetic rats. Hesperidin (100, 200 mg/kg daily) was given to diabetic rats for 12 weeks. The blood-retina breakdown (BRB) was determined after 2 weeks of treatment followed by the measurement of related physiological parameters with ELISA kits and immunohistochemistry staining at the end of the study. Elevated AR activity and blood glucose, increased retinal levels of vascular endothelial growth factor (VEGF), ICAM-1, TNF-α, IL-1β and AGEs as well as reduced retina thickness were observed in diabetic rats. Hesperidin treatment signiﬁcantly suppressed BRB breakdown and increased retina thickness, reduced blood glucose, AR activity and retinal TNF-α, ICAM-1, VEGF, IL-1β and AGEs levels. Furthermore, treatment with hesperidin signiﬁcantly reduced plasma malondialdehyde (MDA) levels and increased SOD activity in diabetic rats. These data demonstrated that hesperidin attenuates retina and plasma abnormalities via anti-angiogenic, anti-inflammatory and antioxidative effects, as well as the inhibitory effect on polyol pathway and AGEs accumulation.

## 1. Introduction

As a leading cause of blindness in middle age and older people, diabetic retinopathy (DR) is one of the most common complications of type 1 and type 2 diabetes [1,2]. Almost everyone with type1 diabetes will develop retinopathy over a 15-20-year period and greater than 60% of type 2 diabetes patients will have retinopathy after 20 years [3]. Diabetic retinopathy, which progresses from nonproliferative abnormalities (increased vascular permeability) to proliferative diabetic retinopathy (growth of new blood vessels), is characterized by retinal edema, hemorrhage, increased neovascularization and neuronal degeneration in the retina [2,4].

Hyperglycemia and poor metabolic control are important factors in the development of diabetic retinopathy [5]. Although the exact mechanism by which hyperglycemia causes vascular disruption in retinopathy is not clear, it has been reported that, in addition to triggering oxidative stress along with inflammatory components [6], hyperglycemia is involved in the development of diabetic retinopathy by increasing the activity of aldose reductase (AR) [7] and protein kinase C (PKC) [8], as well as promoting nonenzymatic glycation and glycooxidation of proteins (AGEs) [9]. Free radicals as reactive oxygen species (ROS) are a strong stimulus for the release of proinﬂammatory cytokines, such as tumor necrosis factor-α(TNF-α) and interleukin 1β (IL-1β), which damage endothelial cells and play an important role in the pathogenesis of DR [10], and it has been reported that anti-inflammatory drugs could prevent early diabetic retinopathy via suppression of proinflammatory cytokines like TNF-α [11]. Besides, it has been reported by Bucolo *et al.* that the up-regulation of proinflammatory factors and angiogenic parameters, such as tumor necrosis factor alpha (TNF-α), vascular endothelial growth factor (VEGF), intercellular adhesion molecule-1 (ICAM-1) and interleukin-1β (IL-1β), contribute to the blood–retinal barrier (BRB) breakdown, which directly leads to macular edema in DR [12,13,14,15]. An important cytokine among the factors involved in the development of diabetic retinopathy is VEGF. VEGF is a cytokine with strong angiogenic and mitogenic actions as a result plays major role in retinal vascular leakage. Besides, VEGF could induce ICAM-1 expression, and inhibition of ICAM-1 activity could signiﬁcantly suppress VEGF-induced hyper-permeability and leukostasis, which indicates that VEGF and ICAM-1 are important mediators in development of DR [16].

**Figure 1:**
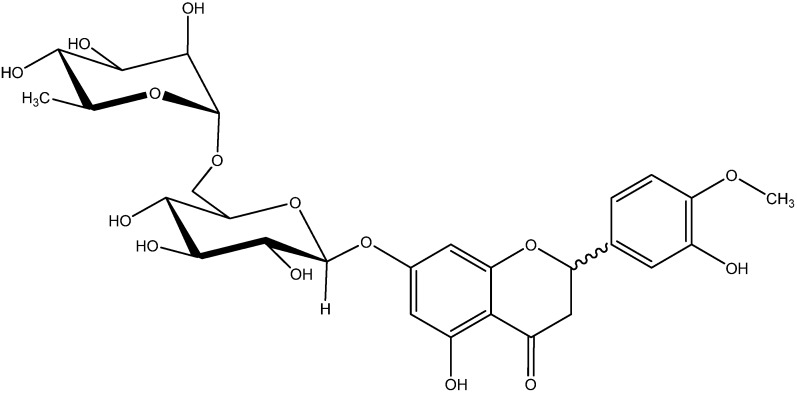
The chemical structure of hesperidin {(±)-2-5-hydroxy-2-(3-hydroxy-4-methoxyphenyl)-7-[(2*S*,3*R*,4*S*,5*S*,6*R*)-3,4,5-trihydroxy-6-[[(2*R*,3*R*,4*R*,5*R*,6*S*)-3,4,5-trihydroxy-6-methyloxan-2-yl]oxymethyl]oxan-2-yl]oxy-2,3-dihydrochromen-4-one}.

It has been reported that supplementation with hesperidin (Hsp, Figure 1), a compound with a flavonone glycoside chemical structure which is abundant in citrus fruits, could significantly suppress oxidative stress in serum, liver, and kidney as well as proinflammatory cytokine production in serum of diabetic rats [17]. Recently it has been demonstrated that hesperidin signiﬁcantly inhibited the high glucose-induced production of ICAM-1 in human umbilical vein endothelial cells (HUVECs) [18]. Besides, it has been confirmed that hesperidin could inhibit the activity of aldose reductase *in vitro* [19]. In addition, in our previous research we found that hesperidin could ameliorate hyperlipidemia in hyperlipemic mice, which is related to the retinal hard exudates in DR [20]. Given the inflammatory and oxidative stress-related nature of DR, we investigated the effects of hesperidin administration on DR elicited by injection of streptozotocin (STZ) in rats and compared the effect of hesperidin to that of calcium dobesilate (CaD). In this study, we evaluated the effects of hesperidin on retinal VEGF, ICAM-1, TNF-α, IL-1β, and AGEs, serum SOD activity and malondialdehyde (MDA) levels and the BRB integrity in STZ-induced diabetic rats. In addition, the effects of hesperidin on the activity of aldose reductase and the retinal thickness in diabetic rats were also determined. In previous researches on the activities of hesperidin, few studies have paid attention to the different configurations of hesperidin. According to previous research, commercially available hesperidin could be easily racemized at the C-2 position, and the hesperidin we used in the experiment, which was purchased from a chemical company, was examined by ^13^C-NMR, and the results (Supplementary Information) showed that the hesperidin was a mixture of (2*S*)- and (2*R*)-hesperidin. This study mainly discussed the effect of the mixture of (2*S*)- and (2*R*)-hesperidin on plasma and retina abnormalities in diabetic rats.

## 2. Results and Discussion

### 2.1. Body Weight and Glycemic Parameters

**Figure 2:**
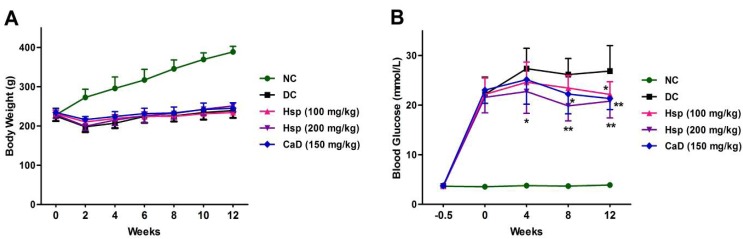
Effects of Hsp and CaD on body weight and blood glucose levels. (**A**) Effects of Hsp and CaD on body weight among different study groups over a duration of 12 weeks in diabetic rats; (**B**) Effects of Hsp and CaD on blood glucose levels over a course of 12 weeks. * *p* < 0.05, ** *p* < 0.01 as compared with the DC group; NC, Normal Control; DC, Diabetic Control. The data represent means ± SD.

As shown in Figure 2, compared with the NC group, the body weights of the DC group were significantly decreased (*p* < 0.05), and the body weight losses were not prevented by treatment with Hsp and CaD (*p* > 0.05). Blood glucose levels were significantly increased in STZ-induced diabetic rats (*p* < 0.01), and treatment with Hsp and CaD led to a significant fall in blood glucose levels (*p* < 0.05).

Diabetic retinopathy is a complication induced by high blood glucose levels, and it has been confirmed by previous researches that the retinal and plasma abnormalities in diabetic rats are similar to that observed in diabetic patients [21,22]. In this study, diabetes was induced by a single injection of STZ [23], which could damage the insulin secreting cells of the pancreas and result in high blood glucose levels that last for a long period. The results showed that the treatment with Hsp and CaD did not change the decreased body weight but significantly lowered the elevated blood glucose levels, though the blood glucose levels are still high above normal levels to cause diabetic complication. 

### 2.2. BRB Breakdown

**Figure 3:**
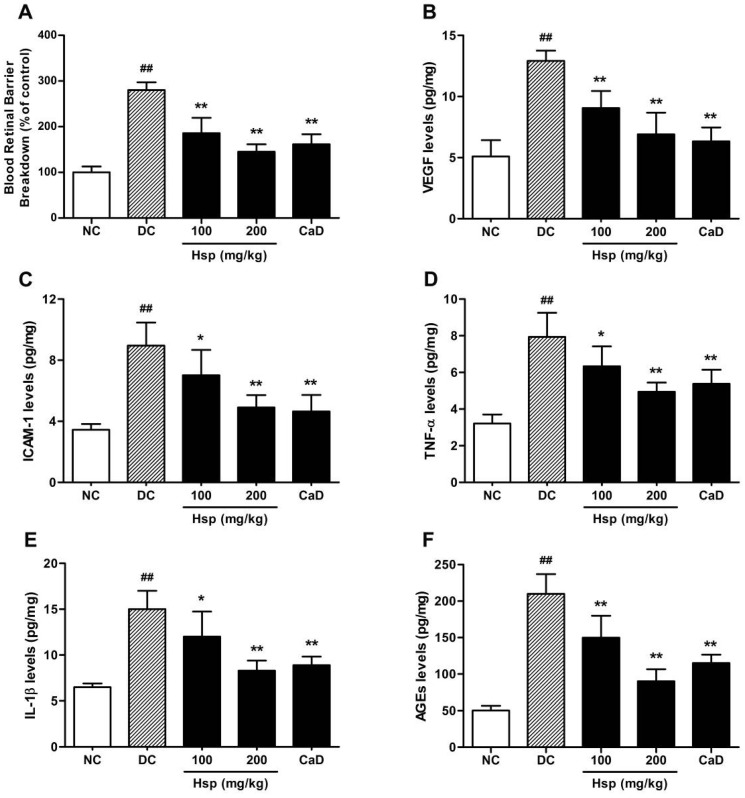
Effects of Hsp and CaD on (**A**) BRB breakdown; retinal levels of (**B**) VEGF; (**C**) ICAM-1; (**D**) TNF-α; (**E**) IL-1β; and (**F**) AGEs in rats. ^##^ *p* < 0.05 as compared with the NC group; * *p* < 0.05, ** *p* < 0.01 as compared with the DC group; NC, Normal Control; DC, Diabetic Control. The data represent means ± SD.

To investigate whether the treatment with Hsp could attenuate the retina abnormalities in the development of DR, we investigated the effects of Hsp and CaD on BRB breakdown first and a dramatic leakage was observed in diabetic rats (Figure 3A Hsp treatment (100 and 200 mg/kg) suppressed diabetes-related BRB breakdown by 34.7% and 48.1%, respectively, compared with diabetic group (*p* < 0.01) while calcium dobesilate treatment suppressed BRB breakdown by 42.3% (*p* < 0.01).

Increased permeability of the BRB, a hallmark of diabetic retinopathy, was observed after 2 and 12 weeks of STZ-induced diabetes in rats as reported previously [24]. The alternation in BRB is closely related to the development of diabetic retinopathy, which is associated with the abnormalities in the secretion of angiogenic and proinflammatory cytokines such as VEGF, ICAM-1, TNF-α and IL-1β [25].

The data (Figure 3A showed that the treatment with Hsp significantly suppressed BRB breakdown and the effect was comparable to that of CaD, which indicated that the treatment with Hsp may be helpful in the treatment of diabetic retinopathy and a 12 weeks study was then carried out to investigate the underlying mechanisms.

### 2.3. Retinal VEGF, ICAM-1, TNF-α, IL-1β, and AGEs Levels

Significantly increased levels of VEGF, ICAM-1, TNF-α, IL-1β and AGEs were observed in retinas of diabetic rats at the end of the study (Figure 3B–F). Treatment with Hsp and CaD signiﬁcantly reduced the levels of retinal VEGF, ICAM-1, TNF-α, IL-1β and AGEs compared with diabetic control group (*p* < 0.01) and the effect of Hsp was comparable to that of CaD (Figure 3B–F). Many studies have identified ﬂavonoids in various foods that are associated with a reduction in the risk of advanced retinal macular degeneration. For example, Bucolo *et al.* found that, eriodictyol, a strong antioxidative flavonoid extracted from *Eriodictyon californicum*, could significantly reduce the retinal levels of VEGF, ICAM-1, TNF-α and eNOS in diabetic rats [14]. Therefore, there is considerable interest in understanding the potential role and benefit of ﬂavonoids in ocular health and disease prevention.

Although various initiators of diabetic retinopathy have been proposed, increased oxidative stress induced by hyperglycemia seems to be the unifying mechanism of diabetic complications, which could activate the polyol pathway, increase AGE formation, activate PKC and hexosamine pathways and lead to the development of DR eventually [26,27]. Free radicals as ROS are also the strong stimulus for the release of proinflammatory cytokines, such as TNF-α and IL-1β, which damage endothelial cells and play an important role in the pathogenesis of DR [10], and it has been reported that anti-inflammatory drugs could prevent early diabetic retinopathy via suppression of proinflammatory cytokines like TNF-α [27]. Among all the cytokines involved in DR, VEGF, which has been identified as a primary initiator of proliferative DR and as a potential mediator of nonproliferative retinopathy [28], is a cytokine with strong angiogenic and mitogenic actions as a result plays major role in retinal vascular leakage. In the retina and elsewhere, VEGF can induce ICAM-1 expression and leucocyte adhesion [29,30], which together with VEGF lead to the BRB breakdown [31], and it has been reported that retinal VEGF and ICAM-1 levels are strongly correlated with neovascularization in patients with DR [32,33].

According to previous research, as the product of nonenzymatic glycation, AGEs is localized to retinal vessels and retinal pericytes. AGEs deposition occurs prior to retinal microvasculature changes and AGEs could colocalize with AGE receptors, induce vascular basement membrane (BM) thickening and lead to upregulation of ICAM-1 and cause BRB breakdown [34].

The data (Figure 3B–F) showed that the retinal levels of VEGF, ICAM-1, TNF-α, IL-1β and AGEs were significantly decreased after 12 weeks treatment with Hsp and CaD compared with diabetic group, and the inhibitory effect of Hsp on AGEs production is more prominent than that of CaD, which suggests that hesperidin may prevent the BRB breakdown and the development of diabetic retinopathy via the anti-angiogenic, anti-inflammatory and antioxidative effects, as well as the inhibitory effect on AGEs accumulation in rat retina.

### 2.4. Aldose Reductase (AR) Activity

**Figure 4:**
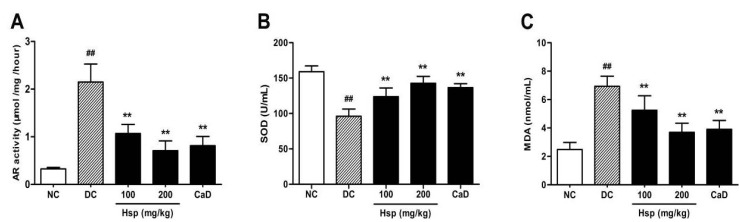
Effects of Hsp and CaD on (**A**) aldose reductase activity in rat eyes; (**B**) SOD activity and (**C**) MDA levels in serum of rats. ^##^ *p* < 0.05 as compared with the NC group; * *p* < 0.05, ** *p* < 0.01 as compared with the DC group; NC, Normal Control; DC, Diabetic Control. The data represent means ± SD.

To investigate the effect of hesperidin on the polyol pathway, we examined AR activity in rat eyes. The results showed that when compared with rats in normal control group, diabetic rats showed signiﬁcantly higher AR activity (*p* < 0.01). Both CaD and Hsp treatments resulted in a signficant decrease in AR activity in treated diabetic rats compared to untreated diabetic rats (*p* < 0.01) (Figure 4A).

As reported in previous studies, AR, as the first enzyme in the polyol pathway, is responsible for the early abnormalities in the pathogenesis of DR, leading to a cascade of retinal lesions including BRB breakdown, loss of pericytes, neovascularization and glial reactivation [7]. Besides, increased AR activity has been shown to contribute to increased oxidative stress by promoting nonenzymatic glycation and the activation of PKC [35].

The result (Figure 4A showed that aldose reductase activity was extremely high in diabetic rats as reported [36]. Treatment with Hsp and CaD both significantly inhibit the elevated AR activity in diabetic rat eyes, which indicates that Hsp may suppress the development of diabetic retinopathy by inhibiting the activation of polyol pathway.

### 2.5. SOD Activity and MDA Levels in Blood

The results showed that compared with rats in the normal control (NC) group, MDA levels in diabetic rats were significantly increased, while SOD activity was significantly decreased at the end of the study (*p* < 0.01, *p* < 0.01), which is consistent with previous research [37]. Compared with untreated rats in the DC group, CaD and Hsp treatments significantly suppressed diabetes-related lipid peroxidation (*p* < 0.01, *p* < 0.01). In addition, the decreased SOD activity in diabetic rats was significantly increased by the treatment with CaD and hesperidin (Figure 4B,C).

It has been reported that the oxidative stress seems to be the unifying mechanism of diabetic complication, and regulation of mitochondrial superoxide levels by SOD attenuates oxidative stress, mitochondrial dysfunction and prevents the development of diabetic retinopathy in mice [38]. As the production of lipid peroxidation, plasma MDA levels have been found to be significantly high in diabetic patients, and MDA may serve as a good marker of oxidative stress in the pathological process [21]. In this study, treatment with hesperidin significantly decreased plasma MDA levels and increased SOD activity compared with DC group, and the effect of hesperidin was comparable to that of CaD, which means that hesperidin may reduce reactive oxygen free radicals and show protective effect in diabetic retinopathy by decreasing oxidative stress.

### 2.6. Histopathological and Immunohistochemical Studies

**Figure 5:**
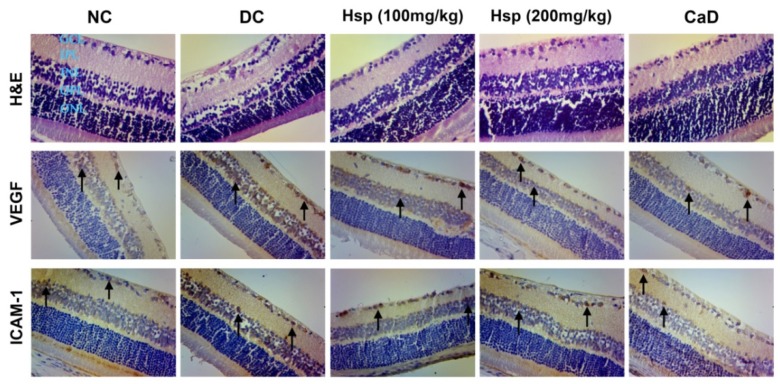
The effects of Hsp and CaD on morphological changes of retinas and expression of VEGF and ICAM-1 in diabetic rat retinas (SP × 200).

**Figure 6:**
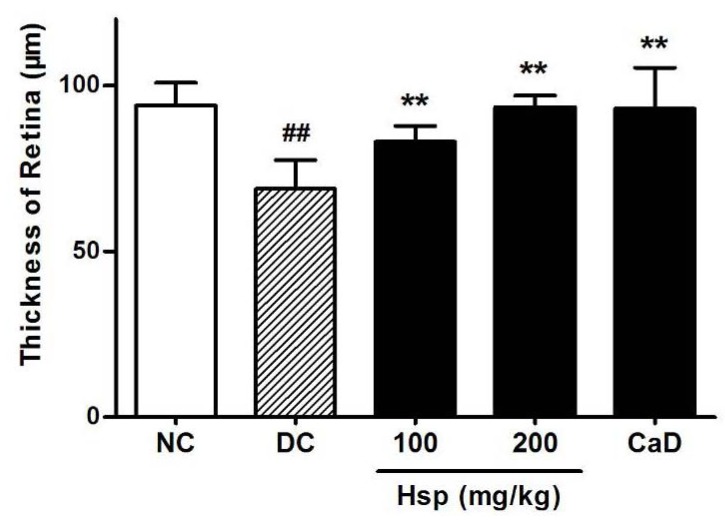
The effects of Hsp and CaD on the thickness of retinas. **^##^** *p* < 0.05 as compared with the NC group; * *p* < 0.05, ** *p* < 0.01 as compared with the DC group; NC, Normal Control; DC, Diabetic Control. The data represent means ± SD.

Morphology study showed that retinal thickness in diabetic rats was significantly reduced compared with that in normal rats (Figure 5), which is in accordance with previous research [39,40]. Although the reason for this change is not clear, the data showed that, compared with DC group, both CaD and Hsp effectively prevented the loss of retinal thickness in treated rats (*p* < 0.01, *p* < 0.01) (Figure 6). As shown in Figure 5, in diabetic rats (DC group), the immunohistochemical staining for VEGF and ICAM-1 revealed that the strong immunoreactivities of VEGF and ICAM-1 were detected in ganglion cell layer (GCL) and inner nuclear layer (INL), while the expression of VEGF and ICAM-1 in NC group showed little brown immunostaining (black arrow). Both Hsp and CaD reduced the expression of VEGF and ICAM-1, which is in accordance with the results determined by ELISA assay.

## 3. Experimental

### 3.1. Chemicals and Drugs

The ELISA kits for measurement of VEGF, ICAM-1, AGEs, IL-1β and TNF-α protein levels were purchased from R&D Co. (Minneapolis, MN, USA). The kits for measurement of blood glucose, superoxide dismutase (SOD) activity and malondialdehyde (MDA) levels were purchased from Jiancheng Bioengineering Institute (Nanjing, China). BCA kit was bought from Beyotime Institute of Biotechnology (Shanghai, China). Evans blue dye, NADPH, DL-glyceraldehyde and streptozotocin (STZ) were purchased from Sigma (St. Louis, MO, USA). Calcium dobesilate (CaD) was purchased from Lijun International Pharmaceutical Co. (Xi’an, China) and hesperidin (purity > 98%) was purchased from Linuo Biotechnology (Zhengzhou, China). All solvents used in this study were of analytical reagent grade.

### 3.2. Animals

Male Sprague-Dawley rats (200 ± 20 g) were purchased from the Qinglongshan Animal Center (Nanjing, China). The animals were housed in standard polypropylene cages (four rats/cage) at 22 ± 2 °C, 45%–75% relative humidity, and 12 h light and 12 h dark cycle. All the rats were provided with normal pellet diet (NPD) (Qinglongshan) and water *ad libitum* for 1 week to acclimatize. All the experimental procedures were performed in accordance with the recommendations in the Guide for the Care and Use of Laboratory Animals of China Pharmaceutical University. The protocol was approved by the Animal Study Committee of China Pharmaceutical University.

### 3.3. Induction of Diabetes

Diabetes was induced by a single injection of streptozotocin (STZ, 60 mg/kg, i.p.) in rats after 12 h of fasting. Age-matched control rats received an equal volume of vehicle (0.01 M citrate buffer, pH 4.5). 72 h after injection, the blood samples were collected from the tail vein and blood glucose levels were measured using OneTouch UltraEasy blood glucose meter (Johnson & Johnson, New Brunswick, NJ, USA). Rats with a blood glucose level over 16.7 mmol/L were considered as diabetes-induced rats. The animals were randomly divided into five groups: (1) normal rats (NC, n = 20), (2) STZ-induced diabetic rats (DC, n = 20), (3) STZ-induced diabetic rats treated with calcium dobesilate, a positive control for treating diabetic retinopathy (DC+CaD, 150 mg/kg body weight, n = 20), (4) STZ-induced diabetic rats treated with hesperidin (DC+Hsp-100, 100 mg/kg body weight, n = 20), (5) STZ-induced diabetic rats treated with hesperidin (DC+Hsp-200, 200 mg/kg body weight, n = 20). Day 3 (72 h) after injection of STZ was designated as day 1 for the treatment of hesperidin and calcium dobesilate in diabetic rats. Hesperidin and calcium dobesilate were administered intragastrically (i.g.) once a day in all rats. 10 rats from each group were sacrificed for the measurement of BRB breakdown at day 14 (2 weeks) of the treatment while other rats were kept and treated with hesperidin and calcium dobesilate for another 10 weeks. At the end of the treatment (12 weeks), blood and retina samples were collected for analysis of AR activity, plasma MDA levels and SOD activity, retinal TNF-α, ICAM-1, VEGF, IL-1β and AGEs levels, as well as histopathological and immunohistochemical studies.The selection of doses was based on preliminary experiments, wherein, doses of 100 and 200 mg/kg were tried and confirmed to be suitable and effective in test rats.

### 3.4. Body Weight and Blood Glucose

The rats were weighed every two weeks and blood samples were collected from the tail vein every 4 weeks since the induction of diabetes and the blood glucose was estimated using OneTouch UltraEasy blood glucose meter (Johnson & Johnson).

### 3.5. Measurement of BRB Breakdown

2 weeks after the treatment begun, 10 rats in each group were anesthetized and BRB breakdown was measured using Evans blue dye (Sigma-Aldrich) injected through the tail vein over 10 s at a dosage of 45 mg/kg [41]. Two minutes after the injection of Evans blue, 0.2 mL blood was drawn from the iliac artery to obtain the initial Evans blue plasma concentration. Subsequently, at 15-minute intervals, 0.1 mL blood was drawn from the iliac artery up to 2 h to obtain the time-averaged Evans blue plasma concentration. 120 min after injection, 0.2 mL blood was drawn from the left ventricle to obtain the final Evans blue plasma concentration. Then, the chest cavity was opened and the rats were perfused via the left ventricle with paraformaldehyde (0.05 M, pH 3.5, 1% (w/v)) at 37 °C for 2 min at a physiological pressure; immediately after perfusion, both eyes were enucleated and the retinas were carefully dissected and thoroughly dried for 5 h. The dry weight was recorded for the quantitation of Evans blue leakage. Evans blue was extracted in 120 μL formamide for 18 h at 70 °C, the supernatant was ultra-centrifuged at a speed of 70,000 rpm for 40 min at 4 °C, and 60 μL of the supernatant was used for triplicate spectrophotometric measurements. A background-subtracted absorbance was determined by measuring each sample at both 620 nm (the absorbance maximum for Evans blue) and 740 nm (the absorbance minimum). The concentration of dye in the extracts was calculated from a standard curve of Evans blue in formamide. BRB breakdown was calculated by Equation (1):

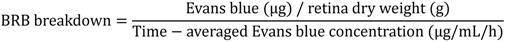
(1)


We expressed results as percentage of non-diabetic controls (NC group).

### 3.6. Retinal VEGF, ICAM-1, TNF-α, IL-1β and AGEs Levels

At the end of treatment, rat eyes were collected and the left ones were used for the measurements of VEGF, ICAM-1, TNF-α, IL-1β and AGEs, each retina was dissected and homogenized in 120 μL of lysis buffer supplemented with protease inhibitors (Beyotime). Samples were centrifuged at a speed of 10,000 rpm for 10 min at 4 °C and the supernatant was used for the determination of VEGF, ICAM-1, TNF-α, IL-1β and AGEs levels with respective ELISA kits. The protein concentration of each sample was assessed with the bicinchoninic acid (BCA) assay. All the measurements were performed in duplicate and the tissue sample concentration was calculated from a stand curve and corrected for protein concentration.

### 3.7. Measurements of SOD and MDA Levels in Serum

At the end of the experiment, blood samples were collected from femoral artery and centrifuged at a speed of 3,500 rpm for 10 min, the serum was used for the determination of SOD and MDA levels according to the instruction of commercial kits.

### 3.8. Aldose Reductase Enzyme (AR) Activity

The right eyes were enucleated and six eyes from each group were homogenized in 1 mL PBS supplemented with 0.5 mM EDTA and 10 mM 2-mercaptoethanol (pH 7.0), then the tissue samples were centrifuged at 5,000 *×g* for 5 min. The pellet was placed into PBS and centrifuged once more (10 min at 4 °C, 25,000 *×g*); the supernatant was used for the measurement of AR activity and the quantity of protein. AR activity was determined spectrophotometrically according to previous research by monitoring the decrease in NADPH absorption at 340 nm at 37 °C using DL-glyceraldehyde as substrate [36]. Unspecific NADPH dehydrogenase activity was recorded for 5 min; then DL-glyceraldehyde was added and the incubation was continued for another 5 min. Values of AR activity given in this study represent the difference between the rate of NADPH oxidation in the presence and absence of substrate (μmol/mg/hour).

### 3.9. Histopathological and Immunohistochemical Studies

Eyes were ﬁxed in 10% formaldehyde and embedded in paraffin, and 4 μm thick sections were prepared. The sections were stained with haematoxylin and eosin. Pictures were taken at both sides of the optic nerve and mid-retina at 200× using a LEICA DM 1000 microscope with MiniSee system. The thickness of retina was measured from inner nuclear layer to inner limiting membrane using Image-Pro Plus 6.0 [40].

Immunohistochemical staining of vascular endothelial growth factor (VEGF) and Intercellular Adhesion Molecule 1 (ICAM-1) was carried out on sections of paraffin-embedded rat eyes. The slides were incubated with the primary VEGF antibody (1:200, US biological, Marblehead, MA, USA) or the primary ICAM-1 antibody (1:100, Santa Cruz, CA, USA) in a humidity chamber overnight. After washing with phosphate-buffered saline, the sections were hybridized with secondary antibodies according to the instruction of MaxVision Kit (Maixin Bio, Fuzhou, China) and colored with DAB, respectively. Images were obtained using an LEICA DM 1000 microscope with MiniSee system. Brown staining in the cytoplasm and/or nucleus was considered an indicator of positive expression.

### 3.10. Statistical Analysis

Data are expressed as means ± SD and *P*-values were determined using Student’s *t*-test (when comparing two groups) or one-way analysis of variance (ANOVA) (when comparing more than two groups). Differences with a value of *p* < 0.05 were considered statistically significant.

## 4. Conclusions

The results of our study showed for the first time that the effect of hesperidin on the abnormalities of physiological parameters closely related with development of DR is prominent. The protective effects of hesperidin on retinal abnormalities could be due to its direct effects on angiogenic parameters and its antioxidant, anti-inflammatory properties, as well as the inhibitory effect on aldose redutase activity and AGEs accumulation in retina, with additional hypoglycemia effect, and the inhibitory effect of hesperidin on AGEs formation in retina is more prominent than the effect of CaD.

Little previous researches on the biological activities of hesperidin took the differences in configuration into consideration. Recently, Daxin *et al.* found that both (2*S*)- and (2*R*)-hesperidin could prevent the formation of AGEs *in vitro* and the activities are not affected by differences in configuration [42], but Brand *et al.* reported that there are some significant differences in the metabolism and transport characteristics between (2*S*)- and (2*R*)-hesperetin, although these differences are relatively small [43]. Taken together, these findings implicate that the differences in the *in vivo* activities of (2*S*)- and (2*R*)-hesperidin are worth thorough investigation. Based on the results in our experiment and well established ocular bioactivity of hesperidin [44], further studies are still needed to explore the exact effect of hesperidin on DR as well as the relationship between configurations and *in vivo* activities.

## Supplementary Materials

Supplementary materials can be accessed at: http://www.mdpi.com/1420-3049/17/11/12868/s1.
