# Supplementary material for: Hesperidin Prevents Retinal and Plasma Abnormalities in Streptozotocin-Induced Diabetic Rats
Source: Molecules. 2012 Nov 1;17(11):12868–81. doi: 10.3390/molecules171112868 (PMC6268103; doi:10.3390/molecules171112868)

Supporting Information

As shown in ^{13}C-NMR spectrum of hesperidin below, the hesperidin we used in the experiment is a mixture of (2*S*)- and (2*R*)-hesperidin.

Figure 1. The ^{13}C-NMR spectrum of hesperidin (DMSO, 125MHz).

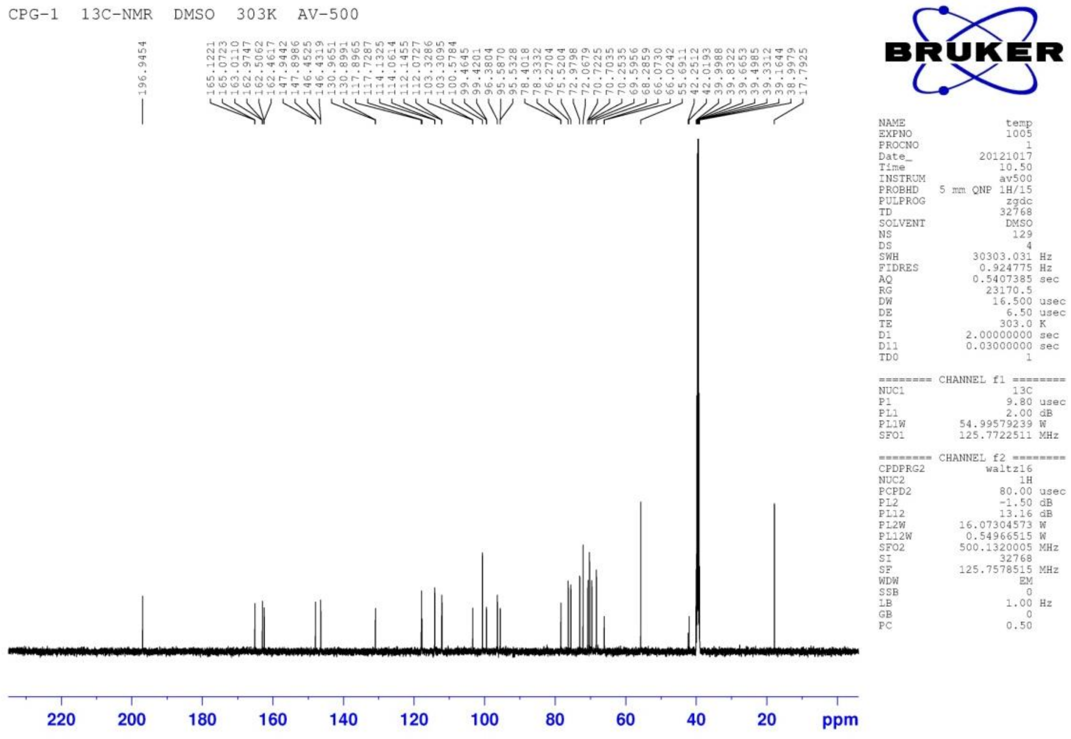

Figure 2. The amplified ^{13}C-NMR spectrum of hesperidin (100–165 ppm).

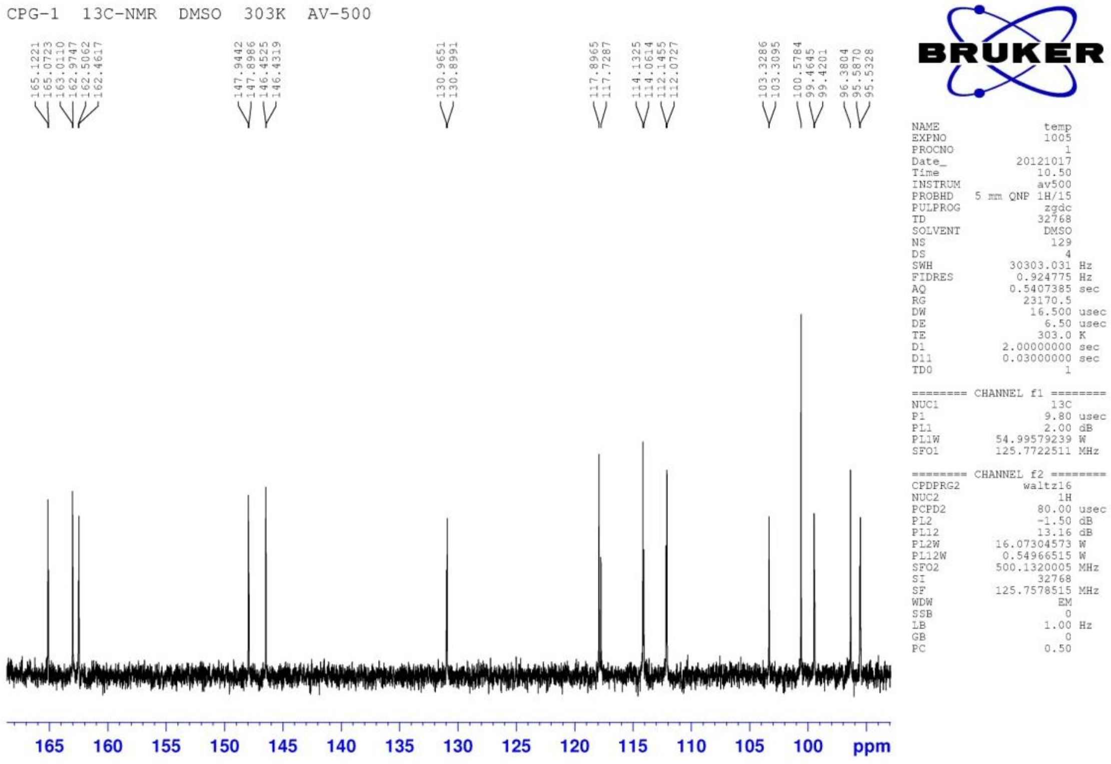

Figure 3. The amplified ^{13}C-NMR spectrum of hesperidin (67–79 ppm).

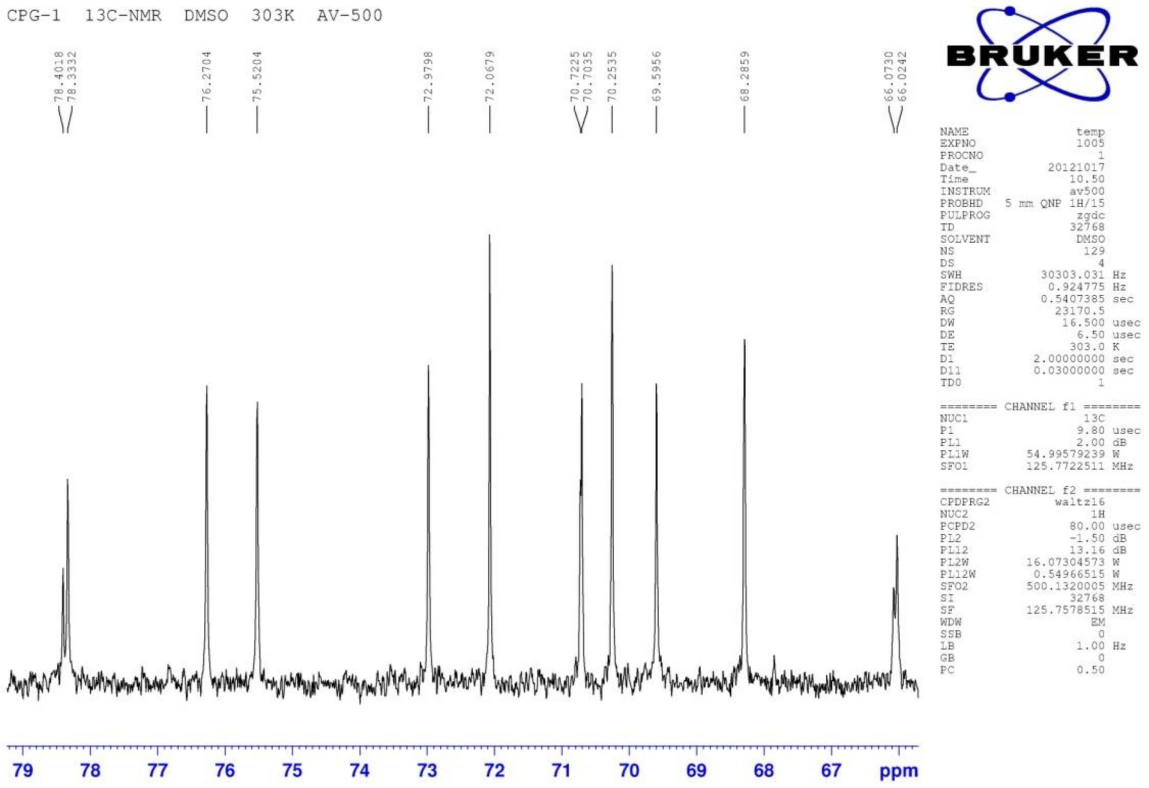

Figure 4. The amplified ^{13}C-NMR spectrum of hesperidin (20–55 ppm).

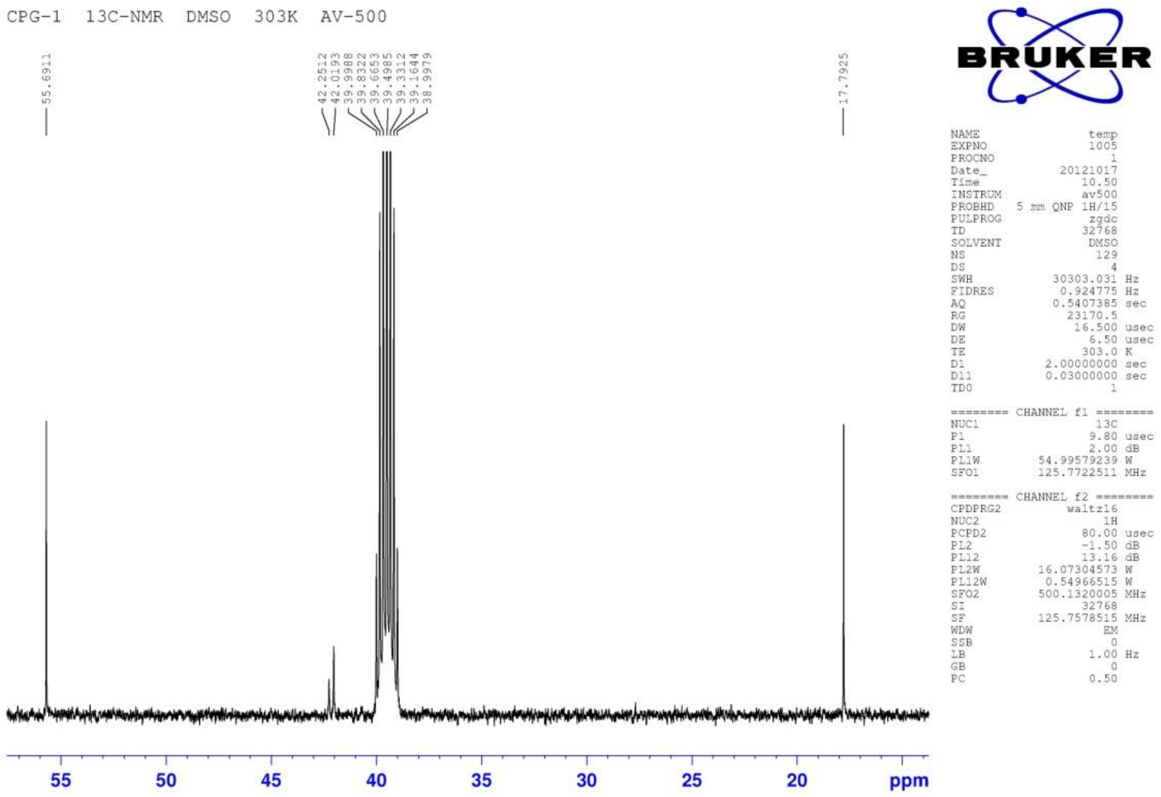

Supplement: Supplementary file 1 [file molecules-17-12868-s001.pdf]
